# Establishment and validation of a model to determine the progression risk of low grade intraepithelial neoplasia

**DOI:** 10.1007/s00464-020-07531-6

**Published:** 2020-05-18

**Authors:** Yuqian Chen, Yini Dang, Huaiming Sang, Xiaoyong Wang, Meihong Chen, Daiwei Lu, Guoxin Zhang

**Affiliations:** 1 Department of Gastroenterology, The First Affiliated Hospital of Nanjing Medical University, Nanjing, China; 2 Department of Gastroenterology, Changzhou NO. 2 People’s Hospital, Changzhou, China; 3 Department of Traditional Chinese Medicine, Nanjing Integrated Traditional Chinese and Western Medicine Hospital Affiliated with Nanjing University of Chinese Medicine, Nanjing, China

**Keywords:** Low-grade intraepithelial neoplasia (LGIN), Early gastric cancer, Risk model

## Abstract

**Objective:**

To establish and validate a model to determine the progression risk of gastric low-grade intraepithelial neoplasia (LGIN).

**Methods:**

A total of 705 patients with gastric LGIN at the endoscopy center of Jiangsu Provincial People's Hospital during January 2010 and August 2017 were retrospectively reviewed. Basic clinical and pathological information were recorded. According to the time sequence of the initial examination, the first 605 patients were enrolled in the derivation group, and the remaining 100 patients were used in the validation group. SPSS 19 software was used as statistical analysis to determine independent risk factors for progression of LGIN of the stomach and to establish a risk model. The ROC was used to verify the application value of the predictive model.

**Results:**

Univariate and multivariate analysis suggested that sex, multiple location, congestion, ulceration and form were independent risk factors for prolonged or advanced progression in patients with LGIN. Based on this, a predictive model is constructed: *P* = ex/(1 + ex) *X* = − 10.399 + 0.922 × Sex + 1.934 × Multiple Location + 1.382 × Congestion + 0.797 × Ulceration + 0.525 × Form. The higher of the *P* value means the higher risk of progression. The AUC of the derivation group and validation group were 0.784 and 0.766, respectively.

**Conclusion:**

Sex, multi-site, hyperemia, ulcer and morphology are independent risk factors for the prolongation or progression of patients with gastric LGIN. These factors are objective and easy to obtain data. Based on this, a predictive model is constructed, which can be used in management of patients. The model can be used to identify high-risk groups in patients with LGIN that may progress to gastric cancer. Strengthening follow-up or endoscopic treatment to improve the detection rate of early cancer or reduce the incidence of gastric cancer can provide a reliable basis for the treatment of LGIN.

Gastric cancer is one of the most common cancers in the world, especially in East Asia. Early diagnosis is the key to improve the survival rate. The development of gastric cancer is a stepwise process, including gastric mucosal inflammation, mild dysplasia, moderate dysplasia, severe dysplasia, and early cancer [1]. The World Health Organization (WHO) introduced the concept of intraepithelial neoplasia (GIN) in a new classification of tumors in 2000 [2, 3]. GIN was classified into low-grade intraepithelial neoplasia (LGIN) and high-grade intraepithelial neoplasia (HGIN) depending on the degree of cell atypia and structural disorder. LGIN includes mild dysplasia and moderate dysplasia, and HGIN includes severe dysplasia and carcinoma in situ. According to the WHO`s new classification of tumors in 2000, LGIN is a precancerous lesion. The clinical treatment guidelines in GIN recommended by WHO/Vierna are as following: (1) conservative treatment: drug treatment and follow-up; (2) endoscopic treatment: (a) resection of the diseased mucosa: endoscopic mucosal resection (EMR) and Endoscopic submucosal dissection (ESD). (b) Damage of the diseased mucosa: The main methods include high-frequency electrocoagulation, argon plasma coagulation, radiofrequency ablation, holmium laser treatment, microwave coagulation therapy, etc. It is recommended by the European guidelines [4] that the found gastric LGIN should be resected and used for a more accurate pathological examination. It is worth noting that the guide emphasizes that the disappearance of LGIN assessed by endoscopic follow-up and biopsy still does not rule out the possibility of progression to aggressive cancer. Depending on the guideline of the American Gastroenterological Association (AGA) and the British Society of Gastroenterology (BSG) [5, 6], endoscopic resection is recommended, regardless of the size of the adenoma and whether there is a combination of dysplasia. The American Society for Gastrointestinal Endoscopy (ASGE) guidelines [5] recommend that the LGIN lesions, which are still found after one year follow-up, should be treated with endoscopic resection. In summary, the different treatment principles are provided in these clinical guidelines because there is currently no suitable solution for the evaluation and management of LGIN worldwide. In China, WHO/Vierna classification is recommended for the treatment of gastric LGIN, such as drug treatment, follow-up or endoscopic treatment. However, there are no uniform criteria accepted to clarify the clinical cases, which are suitable for drug treatment or follow-up, and which require more endoscopic treatment. Endoscopic treatment may not be necessary for some patients with low-grade intraepithelial neoplasia. On the other hand, ignoring the risk of low-grade intraepithelial neoplasia may result in missed diagnosis or misdiagnosis. Furthermore, excessive endoscopic follow-up may increase the risk of examination and the cost of treatment for patients at low risk stage. Therefore, it is necessary and has realistic clinical value to find new methods that can predict and evaluate the likelihood of progression of LGIN.

Here, to analyze the factors associated with the progression of low-grade intraepithelial neoplasia, we constructed a LGIN progression risk model by retrospective study and validated the model. The model is used to predict the prognosis of patients with low-grade intraepithelial neoplasia in order to effectively identify high-risk groups that may progress to gastric cancer. By strengthening monitoring and active treatment, it can reduce the incidence of gastric cancer, and avoid excessive inspection and waste of medical resources.

## Materials and methods

### Research object

A retrospective review of 1011 patients, who underwent gastroscopy at the endoscopy center of Jiangsu Provincial People's Hospital during January 2010 and August 2017 with the age of over 18 years old, were diagnosed as low-grade intraepithelial neoplasia by pathology. The diagnostic criteria refer to the WHO digestive system tumor pathological diagnostic criteria [7]. 54 patients with endoscopic and pathological follow-up records that were absent for more than half a year were recorded. To observe the natural course of low-grade intraepithelial neoplasia, 126 patients who underwent endoscopic ESD or EMR and 17 patients who underwent gastric surgery were excluded. At the same time, 109 patients who underwent endoscopic and pathologically suggesting high-grade intraepithelial neoplasia or gastric cancer within six months were excluded.

### Data collection

Observation indicators: record basic clinical and pathological information, including name, age, gender, outpatient or hospital number, duration of disease, history of gastric surgery, endoscopic findings of lesions (including location, size, morphology, color, phenotype), postoperative histopathological diagnosis and post-stage progression.

Observation of the end point: 1. Pathological examination suggests that the low-grade intraepithelial neoplasia is improved or upgraded. 2. Pathological examination to the end point of the study suggests prolonged intraepithelial neoplasia.

Risk factors were recorded: time course, age, gender, lesion location, multiple sites, lesion size, lesion morphology, lesion color, lesion phenotype, postoperative histopathological diagnosis of intestinal metaplasia as a risk factor for long-term progression.

This study has been approved by the Ethics Committee of the Jiangsu Provincial People's Hospital. The ethical approval number for this study was 2018-SR-276.

### Statistical methods

To build and validate the risk model, we divided the study samples into two groups. According to the time sequence of the initial examination, the first 605 patients were enrolled in the derivation group, and the remaining 100 patients were used in the validation group.

Statistical analysis was performed using SPSS 19 software. Continuity variables were expressed as mean ± standard deviation (SD) and compared by t test. Categorical variables were analyzed using Fisher's exact or chi-square test. Multivariate analysis was performed using a logistic regression model to determine independent risk factors for progression of low-grade intraepithelial neoplasia in the stomach. The receiver operating characteristic (ROC) curve was used to verify the application value of the predictive model. A *P* value < 0.05 was considered statistically significant.

## Result

**Fig. 1:**
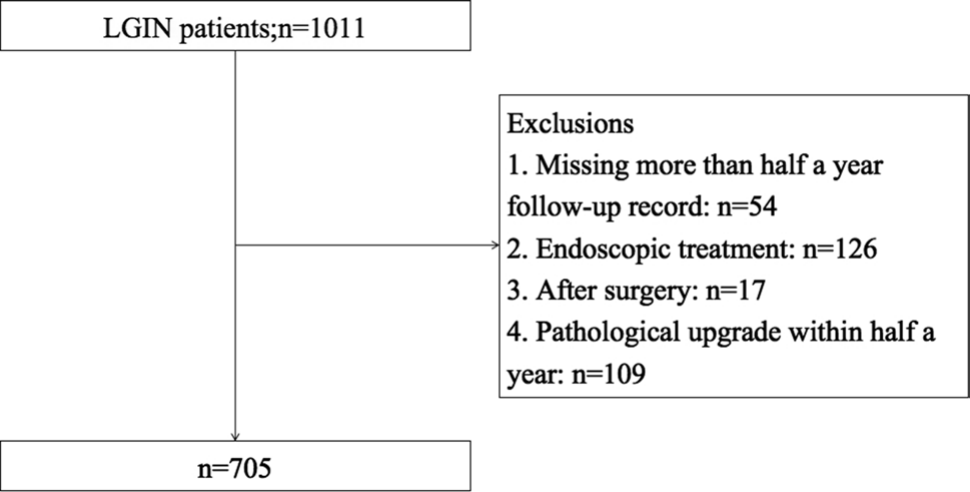
Patient selection for our study

Research Screening Flow Chart (Fig. 1).

### Derived risk model

A total of 605 patients with low-grade intraepithelial neoplasia in the analysis group were analyzed, with an average age of 58.5 ± 10.82 years. The clinical data are shown in Table 1. There were 102 cases without improvement, including 21 advanced progressed cases and 81 prolonged cases. The average time form progression to high tumor or gastric cancer was 3.29 ± 1.92 years, with the 7.28 years at the longest, and 0.95 years at the least. Univariate and multivariate analysis suggested that sex, multiple location, congestion, ulceration and form were independent risk factors for prolonged or advanced progression in patients with low-grade intraepithelial neoplasia. As shown in Table 2.

**Table 1:** Clinical data of patients with low-grade intraepithelial neoplasia in the modeling group

	Total	Retrogress		*P* value
	*n* = 605	Yes *n* = 503	No *n* = 102	
Age (years)				0.404
< 60	337 (55.7%)	284 (56.5%)	53 (52.0%)	
** ≥ **60	268 (44.3%)	219 (43.5%)	49 (48.0%)	
Sex				0.000
Male	373 (61.7%)	294 (58.4%)	79 (77.5%)	
Female	232 (38.3%)	209 (41.6%)	23 (22.5%)	
Lesion location	521	461	60	0.000
Antrum	343 (65.8%)	308 (66.8%)	35 (58.3%)	
Angulus	131 (25.2%)	118 (25.6%)	13 (21.7%)	
Body	13 (2.5%)	13 (2.8%)	0 (0%)	
Fundus	2 (0.4%)	0 (0%)	2 (3.3%)	
Cardia	32 (6.1%)	22 (4.8%)	10 (16.7%)	
Multiple location				0.000
Yes	82 (13.6%)	41 (8.2%)	41 (40.2%)	
No	523 (86.4%)	462 (91.8%)	61 (59.8%)	
Lesion size				0.029
< 2 cm	599 (99.0%)	500 (99.4%)	99 (97.1%)	
** ≥ **2 cm	6 (1.0%)	3 (0.6%)	3 (2.9%)	
Intestinal metaplasia^a^				0.058
0	31 (5.1%)	27 (5.4%)	4 (3.9%)	
I	201 (33.2%)	167 (33.2%)	34 (33.3%)	
II	246 (40.7%)	203 (40.3%)	43 (42.2%)	
III	120 (19.8%)	103 (20.5%)	17 (16.7%)	
IV	7 (1.2%)	3 (0.6%)	4 (3.9%)	
Congestion				0.005
Yes	531 (87.8%)	433 (86.1%)	98 (96.1%)	
No	74 (12.2%)	70 (13.9%)	4 (3.9%)	
Erosion				0.004
Yes	199 (32.9%)	153 (30.4%)	46 (45.1%)	
No	406 (67.1%)	350 (69.6%)	56 (54.9%)	
Nodular surface				0.314
Yes	154 (25.5%)	124 (24.7%)	30 (29.4%)	
No	451 (74.5%)	379 (75.3%)	72 (70.6%)	
Ulceration				0.000
Yes	87 (14.4%)	59 (11.7%)	28 (27.5%)	
No	518 (85.6%)	444 (88.3%)	74 (72.5%)	
Form				0.000
Protuberant	150 (24.8%)	107 (21.3%)	43 (42.2%)	
Flat	452 (74.7%)	394 (78.3%)	58 (56.9%)	
Central Depression	3 (0.5%)	2 (0.4%)	1 (0.9%)	

^a^Intestinal staging according to OLGIM (operative link for gastric intestinal metaplasia assessment) staging [8]

**Table 2:** Univariate analysis and logistic regression analysis

Variable	Univariate analysis		Multivariate analysis	
	OR (95% CI)	*P*	OR (95% CI)	*P*
Sex	2.442 (1.485–4.014)	0.000	2.515 (1.446–4.373)	0.001
Male				
Female				
Multiple Location	7.574 (4.554–12.595)	0.000	6.920 (3.963–12.084)	0.000
Yes				
No				
Lesion Size	5.051 (1.005–25.387)	0.029	1.169 (0.171–7.995)	0.873
< 2 cm				
** ≥ **2 cm				
Congestion	3.961 (1.412–11.107)	0.005	3.981 (1.310–12.095)	0.015
Yes				
No				
Erosion	1.879 (1.218–2.900)	0.004	1.388 (0.854–2.257)	0.186
Yes				
No				
Ulceration	2.847 (1.705–4.755)	0.000	2.218 (1.242–3.963)	0.007
Yes				
No				
Form		0.000	1.690 (1.034–2.763)	0.036
Protuberant	2.697 (1.725–4.219)			
Flat	2.742 (1.756–4.282)			
Central depression	0.403 (0.036–4.489)			

*Constant (*B*) − 10.399

Based on this, build a predictive model: *P* = ex/(1 + ex)

$$X\, = \, - {1}0.{399}\, + \,0.{922} \times {\text{Sex}}\, + \,{1}.{934} \times {\text{Multiple Location}}\, + \,{1}.{382} \times {\text{Congestion}}\, + \,0.{797} \times {\text{Ulceration}}\, + \,0.{525} \times {\text{Form}}.$$

The higher the *P* value, the higher the risk.

**Fig. 2:**
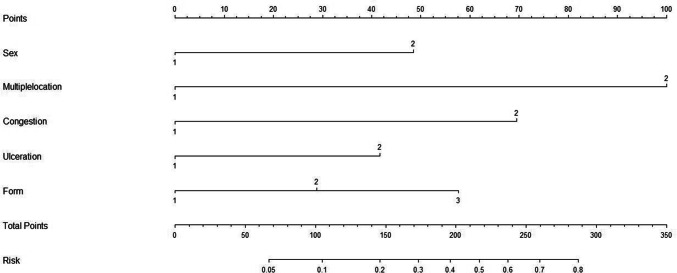
Risk model nomogram

Based on this, the risk model nomogram is drawn, as shown in Fig. 2.

**Fig. 3:**
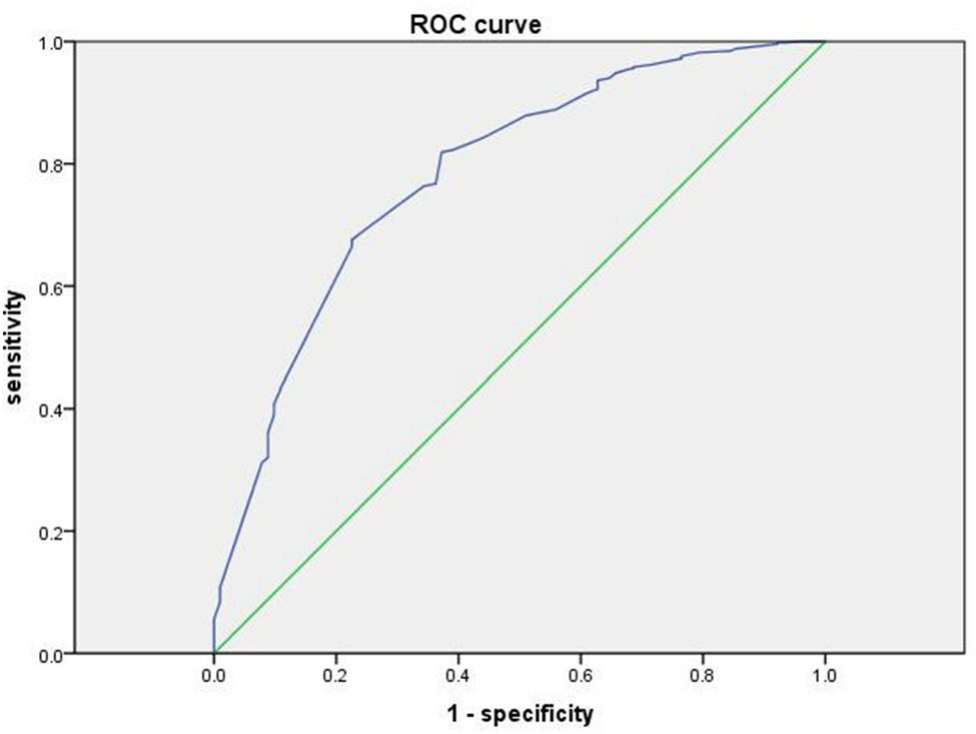
Receiver operating characteristic (ROC) curve of the risk model. AUC = 0.784

The model was constructed based on the clinical data of 605 patients, and the area under the receiver operating characteristic (ROC) curve (AUC) was 0.784, as shown in Fig. 3.

### Verify the risk model

**Fig. 4:**
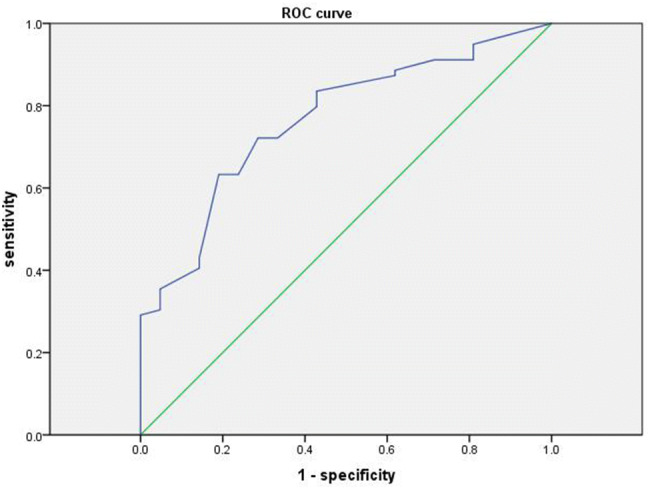
Verifying the receiver operating characteristic (ROC) curve of the case. AUC = 0.766

The clinical data of the remaining 100 patients were verified. The clinical data of the low-tumor patients in the validation group are shown in Table 3. The area under the receiver operating characteristic (ROC) curve (AUC) of 0.766 was applied, as shown in Fig. 4.

**Table 3:** Clinical data for patients with low tumors in the validation group

	Total	Retrogress		*χ*^2^
	*n* = 100	Yes *n* = 79	No *n* = 21	
Age (years)				5.351
< 60	51 (51%)	45 (57.0%)	6 (28.6%)	
** ≥ **60	49 (49%)	34 (43.0%)	15 (71.4%)	
Sex				4.501
Male	72 (72%)	53 (67.1%)	19 (90.5%)	
Female	28 (28%)	26 (32.9%)	2 (9.5%)	
Lesion location	86	71	15	23.658
Antrum	45 (52.3%)	39 (54.9%)	6 (40%)	
Angulus	24 (27.9%)	20 (28.2%)	4 (26.7%)	
Body	5 (5.8%)	3 (4.2%)	2 (13.3%)	
Fundus	1 (1.2%)	1 (1.4%)	0 (0%)	
Cardia	11 (12.8%)	8 (11.3%)	3 (20%)	
Multiple location				4.688
Yes	14 (14%)	8 (10.1%)	6 (28.6%)	
No	86 (86%)	71 (89.9%)	15 (71.4%)	
Lesion size				1.145
< 2 cm	95 (95%)	76 (96.2%)	19 (90.5%)	
** ≥ **2 cm	5 (5%)	3 (3.8%)	2 (9.5%)	
Intestinal metaplasia				
0	8 (8%)	7 (8.9%)	1 (4.8%)	5.238
I	37 (37%)	30 (38.0%)	7 (33.3%)	
II	38 (38%)	32 (40.4%)	6 (28.6%)	
III	17 (17%)	10 (12.7%)	7 (33.3%)	
IV	0 (0%)	0 (0%)	0 (0%)	
Congestion				1.595
Yes	87 (87%)	67 (84.8%)	20 (95.2%)	
No	13 (13%)	12 (15.2%)	1 (4.8%)	
Erosion				1.558
Yes	36 (36%)	26 (32.9%)	10 (47.6%)	
No	64 (64%)	53 (67.1%)	11 (52.4%)	
Nodular surface				0.743
Yes	26 (26%)	19 (24.1%)	7 (33.3%)	
No	74 (74%)	60 (75.9%)	14 (66.7%)	
Ulceration				4.688
Yes	14 (14%)	8 (10.1%)	6 (28.6%)	
No	86 (86%)	71 (89.9%)	15 (71.4%)	
Form				12.431
Protuberant	36 (36%)	23 (29.1%)	13 (61.9%)	
Flat	63 (63%)	56 (70.9%)	7 (33.3%)	
Central depression	1 (1%)	0 (0%)	1 (4.8%)

## Discussion

Endoscopic ESD therapy is recommended without exception for the pathological diagnosed HGIN in domestic and international guidelines. However, the principle of LGIN treatment has been controversial. It is worth noting that LGIN is a precancerous lesion. Although with a low chance of progressing to cancer, it is reported that there is a 0–23% progression rate in HGIN, and the annual rate of gastric cancer progression in LGIN is around 0.6% [9]. Another 10-year follow-up study indicated that 49.4% of the LGIN were reversed, 18.5% of patients with LGIN remained unchanged for a long time, and 32.1% of patients with LGIN developed, of which approximately 17.3% developed advanced gastric cancer [10]. Therefore, the risk of LGIN progressing to gastric cancer cannot be ignored. The progressing of LGIN is slow, with the average time for LGIN progressing to cancer is 10 months to 4 years [11, 12]. Although the treatment of LGIN is inconsistent, long-term follow-up is almost recommended by all guidelines. This recommendation exacerbates the patient's financial burden and potential medical risks. Therefore, it is extremely important to establish a LGIN progress risk model. Using this model, the LGIN population with low risk of cancer (no need for regular checkups), the middle-risk LGIN population (requires regular checkups), and the high-risk LGIN population (requiring short-term treatment) can be identified.

We found that there is no significant correlation between LGIN progression and age, which is consistent with previous studies [13–15]. Man is an independent risk factor for prolonged or progression of low-grade intraepithelial neoplasia, which is consistent with the higher incidence of gastric cancer in men [16–18]. Interestingly, multiple site onset as an independent risk factor for LGIN progression is contrary to the common conclusions of single lesions in gastric cancer. Hou et al. [19] reported that LGIN of the surface with hyperemia and surface ulcer may progress to high-grade intraepithelial neoplasia or early cancer. Another earlier study [20] also confirmed that the structural of gastric mucosa changes following the LGIN lesions progressed. Central depression or nodular surface is associated with progression of LGIN lesions. In this study, it was found that LGIN prolongation or progression was associated with surface redness, lesion size, erosion, morphology (flat, bulging, central depression) or ulceration in a univariate analysis. It showed that there was no significant correlation between the prolongation or progression of LGIN and the size of the lesion or the surface of the nodule by multivariate analysis. The prolongation or progression of LGIN is only associated with the factors, such as lesion size, nodular surface, reddish surface, morphology (flat, bulging, central depression) or ulceration. This may be related to the reduction in the number of related cases undergo endoscopic treatment due to pathological diagnosis inconsistency in cases with lesions > 2 cm or nodular surface. A large number of data show that the diagnosis results of LGIN from gastroscopy biopsy are different from the results from the large biopsy. It has been reported [21] that the pathological difference between endoscopic forceps biopsy and surgical resection is 20.1%. The surface diameter over than 1 cm, surface redness and nodular surface are significant risk factors. Ryu et al. [22] showed that the central depression, nodular surface and surface redness were significantly associated with ECG and low-grade dysplasia lesions. Therefore, the reduction of LGIN cases may be due to the inconsistent diagnosis. It is believed that size is a common feature of malignant tumors [23]. As the size of the lesion increases, the risk of progression of LGIN increases. Another study showed [24] that the prognosis of gastric mucosal LGIN is related to the morphology of endoscopic lesions. The multiple proliferative lesions had the highest rate of regression, while the ulcerated lesions had a higher rate of progression. About 25.42% of the ulcerated lesions progressed to HGIN and gastric cancer, which is consistent with our results. OLGA/OLGIM staging is currently a method for assessing the accuracy of gastric mucosal atrophy/intestinal metaplasia. OLGA/OLGIM III and IV are high-risk patients with gastric cancer [8, 25]. Compared with OLGA, the OLGIM staging system has a higher interdisciplinary diagnostic agreement rate, and lower sensitivity [26, 27]. In this study, we found that intestinal metaplasia was not an independent risk factor for progression of LGIN, which may be related to the assessment of intestinal metaplasia. Wu et al. [13] found that the LGIN intestinal metaplasia rate in the stomach corner is significantly higher than that in the cardia. In contrast, the cancer rate in the stomach corner is much lower than that in the sacral region. It is proved that intestinal metaplasia is not necessarily related to the progression of LGIN, which is consistent with our results.

The development of gastric cancer is a stepwise process, including gastric mucosal inflammation, mild dysplasia, moderate dysplasia, severe dysplasia, and early cancer [1]. Low-level intraepithelial neoplasia is a state in this transformation process. However, as a retrospective study, the study object was to review the cases that were followed up at the Endoscopic Center of Jiangsu Provincial People's Hospital during January 2010 and August 2017. The follow-up time was less than 7.67 years. A 10-year follow-up study indicated that 18.5% of patients with LGIN remained unchanged for a long time [10].We recorded deferred and progressed cases as non-improved groups. By the end of the study, more than two-thirds of the patients in the non-improved groups were still in a prolonged state, and we could not determine their final outcome. Therefore, the cutoff value calculated from this data should be inaccurate, and we may need further follow-up to get a more accurate result.

Today, global research on gastric cancer is focused on the identification and management of HGIN. But there are few studies on LGIN. Here, we focused on the study of early gastric cancer in the target population of LGIN. The duration of disease, age, gender, lesion location, multi-site, lesion size, lesion morphology, lesion color, lesion phenotype, and postoperative histopathological diagnosis of intestinal metaplasia were selected as risk factors for low-grade and long-term progression. These factors are more objective and easily accessible, with highly feasible in clinical use. In this study, patients who underwent endoscopy and pathology for HGIN or gastric cancer within six months were excluded. Therefore, cases in which the pathological diagnosis of biopsy is inconsistent with the pathological diagnosis of gross specimens are excluded. Patients who underwent endoscopic treatment were also excluded. This allows us to observe the natural course of LGIN development. As far as we know, this kind of study has not been reported in the previous studies. Based on this, we established a LGIN Progress Risk Model to identify high-risk groups that may progress to gastric cancer. Strengthening follow-up or endoscopic treatment of the groups can improve the detection rate of early cancer or reduce the incidence of gastric cancer, and provide a reliable basis for the treatment of LGIN. However, this study is a retrospective study and inevitably there will be selective bias in the sample. In addition, in view of China's national conditions, a large number of patients with LGIN have not been reviewed, and may also have an impact on the results of the study.
